# Genome-Wide Scan for Copy Number Alteration Association with Relapse-Free Survival in Colorectal Cancer with Liver Metastasis Patients

**DOI:** 10.3390/jcm7110446

**Published:** 2018-11-18

**Authors:** Po-Sheng Yang, Hsi-Hsien Hsu, Tzu-Chi Hsu, Ming-Jen Chen, Cin-Di Wang, Sung-Liang Yu, Yi-Chiung Hsu, Ker-Chau Li

**Affiliations:** 1 Department of Medicine, Mackay Medical College, New Taipei 252, Taiwan; psyangd0039@gmail.com; 2 Department of General Surgery, Mackay Memorial Hospital, Taipei 104, Taiwan; 3 Department of Colorectal Surgery, Mackay Memorial Hospital, Taipei 104, Taiwan; hsu5936@ms3.hinet.net (H.-H.H.); tzuchi@ms2.mmh.org.tw (T.-C.H.); mjchen@ms1.mmh.org.tw (M.-J.C.); 4 Institute of Statistical Science, Academia Sinica, Taipei 115, Taiwan; samaya6529@gmail.com (C.-D.W.); kcli@stat.sinica.edu.tw (K.-C.L.); 5 Department of Clinical Laboratory Sciences and Medical Biotechnology, College of Medicine, National Taiwan University, Taipei 100, Taiwan; slyu@ncu.edu.tw; 6 Department of Biomedical Sciences and Engineering, National Central University, Taoyuan 320, Taiwan; 7 Department of Statistics, University of California Los Angeles, Los Angeles, CA 90095, USA

**Keywords:** colorectal cancer liver metastases, copy number alteration, gene signature, relapse-free survival, biomarker

## Abstract

Predicting a patient’s risk of recurrence after the resection of liver metastases from colorectal cancer is critical for evaluating and selecting therapeutic approaches. Clinical and pathologic parameters have shown limited accuracy thus far. Therefore, we combined the clinical status with a genomic approach to stratify relapse-free survival in colorectal cancer liver metastases patients. To identify new molecular and genetic signatures specific to colorectal cancer with liver metastasis (CRCLM) patients, we conducted DNA copy number profiling on a cohort of 21 Taiwanese CRCLM patients using a comparative genomic hybridization (CGH) array. We identified a three-gene signature based on differential copy number alteration between patients with different statuses of (1) recurrence and (2) synchronous metastasis. In relapse hotspot regions, only three genes (*S100PBP*, *CSMD2*, and *TGFBI*) were significantly associated with the synchronous liver metastasis factor. A final set of three genes—*S100PBP*, *CSMD2*, *TGFBI*—significantly predicted relapse-free survival in our cohort (*p* = 0.04) and another CRCLM cohort (*p* = 0.02). This three-gene signature is the first genomic signature validated for relapse-free survival in post-hepatectomy CRCLM patients. Our three-gene signature was developed using a whole-genome CGH array and has a good prognostic position for the relapse-free survival of CRCLM patients after hepatectomy.

## 1. Introduction

Colorectal cancer (CRC) is the third most common human malignancy worldwide and the third leading cause of cancer death in the U.S. [1,2]. In Taiwan, the number of new cases of CRC has increased every year, becoming the most and second most prevalent form of cancer in males and females, respectively [3]. Despite the improvement of early diagnostics, synchronic metastasis was noted in about 20%–25% of CRC patients at diagnosis, and these patients had a less than 10% 5-year survival rate [4]. About 50% of CRC patients developed liver metastasis after treatment of their primary tumors, and approximately one-third of these metachronous patients had the disease confined to the liver [5,6]. The median survival was about 5–10 months for CRC patients with liver metastasis without treatment, and less than 0.5% of these patients survived beyond 5 years [7]. For colorectal cancer with liver metastasis (CRCLM), hepatic resection remains the only option with the potential to cure. However, only 15%–25% of CRCLM patients are cured, and 70% experience tumor recurrence [8,9]. Perioperative systemic therapy is usually suggested in patients with resected CRCLM, but a large randomized controlled trial showed that there was no improvement in 5-year overall survival (OS) compared to patients treated with hepatic resection alone (51% vs. 48%) [10]. Almost 30% of patients died with cancer within 2 years after surgery for CRCLM [10], and the selection of optimal treatments for metastatic colorectal cancer is still a complex issue. Therefore, the development of the new molecular and genetic signatures to identify patients at a high risk of relapse after hepatectomy for CRCLM is important.

Many studies have shown that DNA copy number alteration (CNA) correlates with outcome in colon cancer patients [11,12,13,14], and somatic CNA is crucial for the development of CRC [15]. Tumor metastasis is a complex process, and the series of molecular events leading to metastasis is still unclear [16,17,18,19,20]. While several studies have focused on genetic heterogeneity in the many primary malignancies of CRCs, there is consensus on the genetic heterogeneity between a primary cancer and its distant metastasis [21,22,23]. Furthermore, no prognostic CNA signatures have been developed to assess outcomes after hepatectomy for CRCLM.

To identify new molecular and genetic signatures specific to CRCLM patients, we conducted DNA copy number profiling on a cohort of 21 Taiwanese CRCLM patients by a comparative genomic hybridization (CGH) array. We identified a three-gene signature associated with cancer recurrence which was prognostic for relapse-free survival in our cohort. We externally validated this signature in a public cohort of 45 patients after hepatectomy for CRCLM. Herein, we identify a three-gene signature that is prognostic for relapse-free survival and present the validation results of this signature in an independent cohort.

## 2. Materials and Methods

### 2.1. Patient Samples

We obtained 21 paired normal liver and CRCLM metastatic tissues of patients from the anonymized specimens deposited in the Mackey Memorial Hospital tissue bank, in accordance with the protocol approved by the Mackey Memorial Hospital’s Institutional Review Board (13MMHIS009).

### 2.2. Array CGH and Data Processing

The whole-genome NimbleGen CGH array (NimbleGen^®^; NimbleGen Systems Inc, Madison, WI, USA) containing 385,806 probes with spacing of around 6000 bp was used for comparative genomic hybridization of DNA from frozen cancer tissues compared to normal DNA extracted from the PBMC of one male and one female in a community cohort. Patient DNA was required to pass a quality check by agarose electrophoresis. Digital sonifier (Branson Model#450, Branson, Danbury, CT, USA) was used for DNA fragmentation. Labeling, hybridization, and washing were performed according to the manufacturer’s protocol. Array scanning and image generation were performed by the GenePixTM Reader (Personal 4000B, Axon Instruments, Molecular Devices, Sunnyvale, CA, USA) and GenePix^®^ Pro 6.0 software (Axon Instruments, Union City, CA, USA). Generation of log-intensity ratio data with normalization was performed by NimbleScanTM version 2.4 (Roche Nimblegen, Madison, WI, USA) and SignalMapTM version 1.9 software (Roche Nimblegen Madison, WI, USA). For signal enhancing, elementary blocks were formed from the original 385,806 probes in the array by grouping 10 consecutively located probes together [24]. Visualization of CNA profiles was performed by applying the GLAD algorithm (R package from Bioconductor, ). We computed the t-test-significant probe density and produced a density bar plot by the CIRCOS rings. The CIRCOS program was downloaded from http://circos.ca/. The CGH array and clinical data were submitted to the Gene Expression Omnibus archive, available under accession number GEO: GSE103088.

### 2.3. Statistical Analysis

Continuous variables are expressed as the mean ± SD or median (range) according to their homogeneity. Demographic and clinical variables were analyzed by Fisher’s exact test and multivariable logistic regression. Statistical significance was defined as a two-tailed *p* value < 0.05. Fisher’s exact test and logistic regression were accomplished using R version 3.3.1 (https://www.r-project.org/).

### 2.4. Functional Enrichment Analysis of Synchronous Metastasis-Associated Genes

We use the Ingenuity Pathway Analysis (IPA) (QIAGEN company, Redwood City, CA, USA), a web-based computational platform, to conduct functional enrichment analysis of genes. We input the set of 119 synchronous metastasis-associated genes (273 probes) and used the Core analysis enrichment tool with the default settings.

### 2.5. Validation Cohort

Genome-wide copy number analysis was performed on 45 patients with metastatic colorectal cancer using the Affymetrix SNP 6.0 Array [25]. We used the copy number variation of the first metastasized part in each patient and associated it with the overall and relapse-free survival (GSE63490).

### 2.6. Survival Analysis

A patient’s risk score was calculated as the sum of the levels of copy number variation of each gene. Patients were classified as having a high-risk gene signature or a low-risk gene signature, with the median of the risk score as the threshold value. Survival curves for both groups were obtained by the Kaplan–Meier method and were compared using the log-rank test. Both univariate and multivariate Cox regression models were applied for prediction of patient survival. Both the log-rank test and the Cox test were two-sided, and a *p*-value < 0.05 was considered statistically significant.

## 3. Results

### 3.1. Patient Demographics

A total of 21 CRCLM patients between December 2009 and December 2011 were included in the study, comprising 12 colon and 9 rectal cancer patients. Ages ranged from 39 to 83 years old (mean 60.9 years old) with 12 males and 9 females. Follow-up times ranged from 14.5 months to 55.7 months with a mean of 30.2 months. The primary CRC tumor size ranged from 1.2 to 8 cm. Thirteen CRCLM patients were synchronous while, of the eight metachronous patients, three had initial stage II and five had initial stage III. All metachronous CRCLM patients received adjuvant chemotherapy after hepatectomy. Two recurrences were noted in the initial stage II metachronous CRCLM patients and three recurrences were noted in the initial stage III metachronous CRCLM patients. Neoadjuvant chemotherapy was performed in all eight synchronous CRCLM patients before liver resection. All synchronous CRCLM patients received adjuvant chemotherapy after hepatectomy. All eight synchronous CRCLM patients who received neoadjuvant chemotherapy before hepatectomy experienced recurrence (six in the liver and four distant metastasis), but recurrence did not happen in the other five synchronous CRCLM patients who did not receive neoadjuvant chemotherapy before hepatectomy. In total, 13 out of 21 patients died in this study (Table 1). The copy number variations with these clinical variables are displayed in the circos plot in Figure S1.

### 3.2. Genome-Wide Copy Number Alteration (CNA) Profiles for 21 Patients with Colorectal Liver Metastases

**Figure 1:**
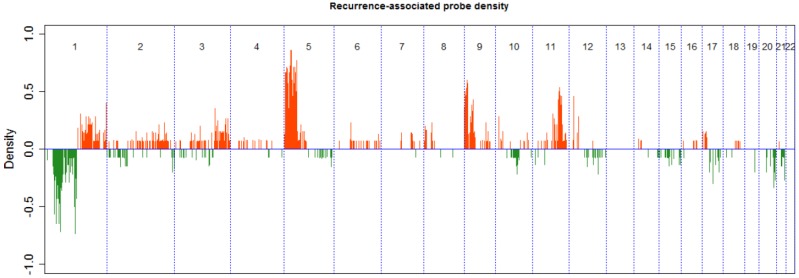
Frequency plots of DNA copy number aberrations in 8 patients without recurrence and 13 patients with recurrence.

The genome-wide CNA profiles for 21 patients with colorectal liver metastases are shown in Figure 1. Frequency plots (Figure 1) indicate that recurrence-associated regions were enriched on chromosome 1 and chromosome 5 (Fisher’s exact probability test; *p* = 1.76 × 10^−17^, 4.32 × 10^−34^, respectively, Table S1). In addition, we focused on regions significantly correlated with synchronous liver metastasis and identified three synchronous metastasis-associated genes in the relapse-associated hotspot regions chr1 and chr5.

### 3.3. Signature Identification

**Figure 2:**
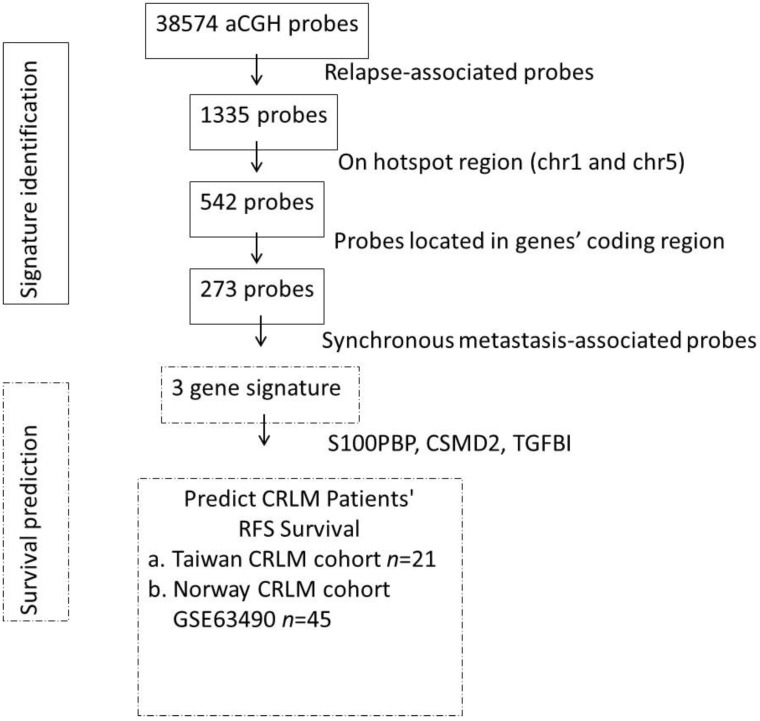
Flowchart of gene selection and analysis procedures.

**Figure 3:**
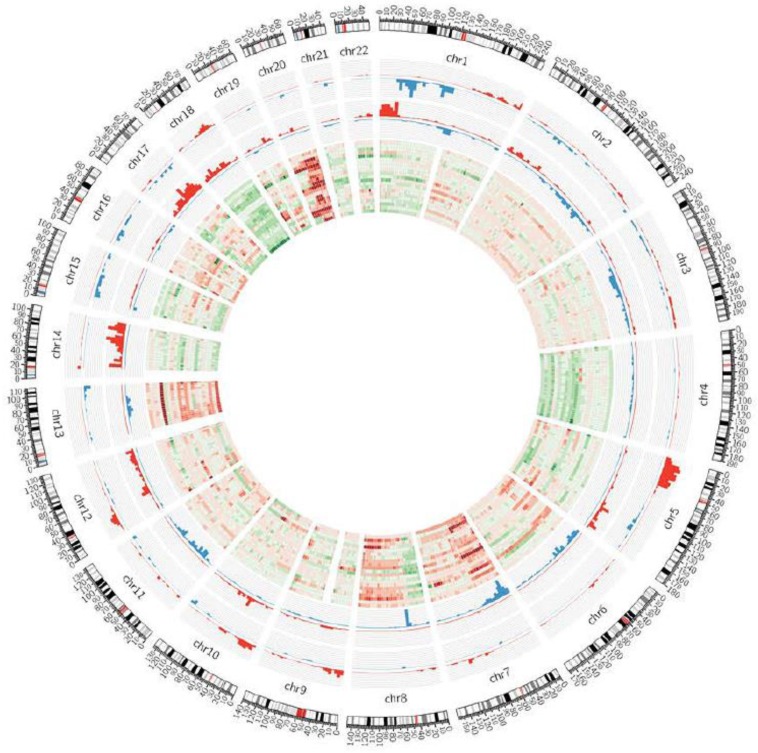
The circos plot of relapse hotspot regions in the CRCLM cohort. Outside to inside: synchronous metastasis (yes vs no); recurrence (yes vs no); copy number alteration in 21 CRCLM patients.

**Figure 4:**
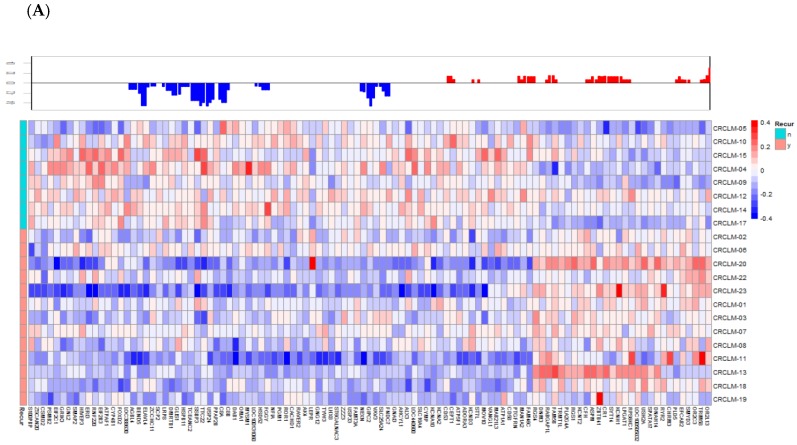

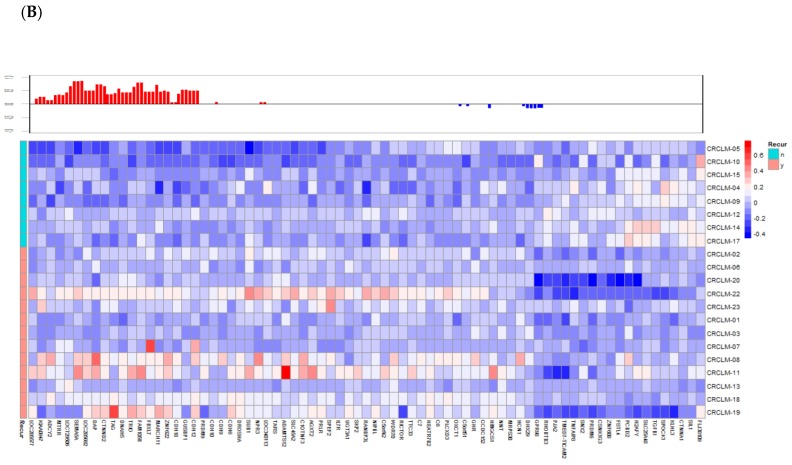
(**A**) The recurrence-associated region on chromosome 1. (**B**) The recurrence-associated region on chromosome 5.

Through a series of statistical analyses to combine information from copy number alterations, cancer recurrence, and synchronous metastasis status, a gene signature was obtained. We identified the genes involved in the colorectal cancer metastasis signaling pathway, such as *CTNNB1*, *WNT*, *JAK*, and *AKT* (Figure S2). Figure 2 outlines the gene selection and data analysis procedures we performed. First, we compared the CNA profiles between 13 relapsed and 8 non-relapsed patients and found 1335 probes with significant differences. These 1335 relapse-associated probes were used for further statistical analyses. We inspected the distribution of relapse-associated probe regions on each chromosome. The results are shown in Table S1 along with the total number of gene-harboring probes, the frequency and density of t-test-significant probes, as well as the results of Fisher′s exact test. Two hotspot regions were enriched on chromosome 1 and chromosome 5 (Table S1). In the corresponding hotspot regions in Figure S1, we identified 542 probes in relapse-association hotspots (chr1 and chr5) and found 273 probes located in gene regions. Synchronous liver metastases are found in around 25% of patients at the time of colorectal cancer diagnosis, which is limited to the liver in 30% of patients [26]. We focused on two clinical variables—recurrence states (yes vs no) and synchronous metastasis (yes vs no)—and we used Students′ t-test to identify the probes with differential CNAs between patients with different statuses of two clinical variables: recurrence states (yes vs no) and synchronous metastasis (yes vs no). The t-test-significant probes are presented by the density bar shown in the circos plot (Figure 3). We also determined that chromosome 20 had significant amination and chromosome 18 had significant depletion in metastasized colorectal cancer. Chromosome 20 amplification has been demonstrated in colorectal cancers with liver metastasis [27,28] and loss of heterozygosity in the 18q region has been found in colorectal cancer [29]. To investigate the recurrence-associated candidate genes of CRCLM, we started from the enriched chromosomes of the recurrence-associated probe blocks. There were 542 recurrence-associated probes on chr1 and chr5. We further selected those probe blocks that were also associated with synchronous metastasis, and a final set of three genes (*S100PBP*, *CSMD2*, and *TGFBI*) were obtained. The heatmap for the correlations between gene copy number variation and patients′ recurrence status on chromosome 1 and 5 showed that most probes were associated with a loss of copy number in the recurrence group (Figure 4).

### 3.4. Biological Assessment In Silico Approach

**Figure 5:**
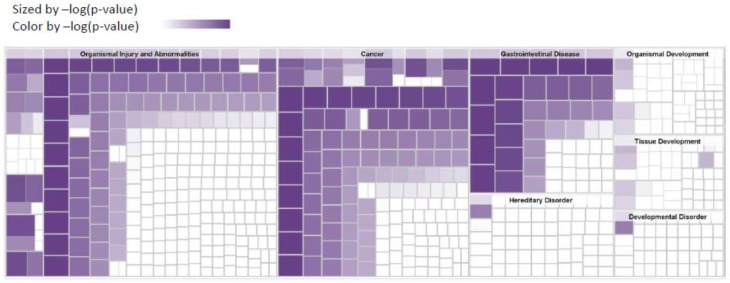
Heatmap for expression analysis in disease and functions.

We use the IPA for diseases and biological functions to conduct a functional enrichment analysis of synchronous metastasis-associated genes. The results show that our genes were enriched in cancer (*p* = 1.24 × 10^−9^), gastrointestinal disease (*p* = 1.24 × 10^−9^), and hepatic systems disease (*p* = 5.27 × 10^−9^) (Figure 5). The most significant disease and disordered biological functions associated with synchronous metastasis genes were related to cancer, organismal injury, and gastrointestinal disease. We found that the top four significant diseases were associated with CRCLM (Table 2).

### 3.5. Clinical Outcome Prediction in CRCLM Patients 

**Figure 6:**
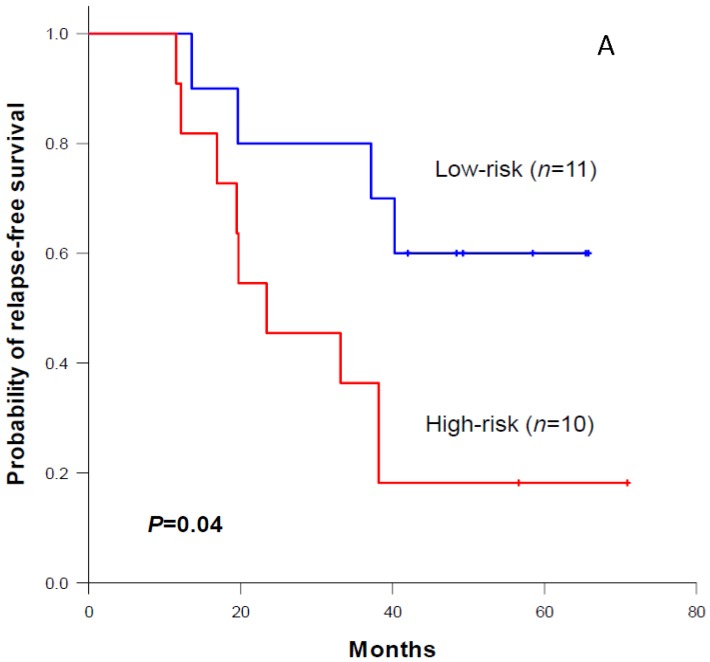

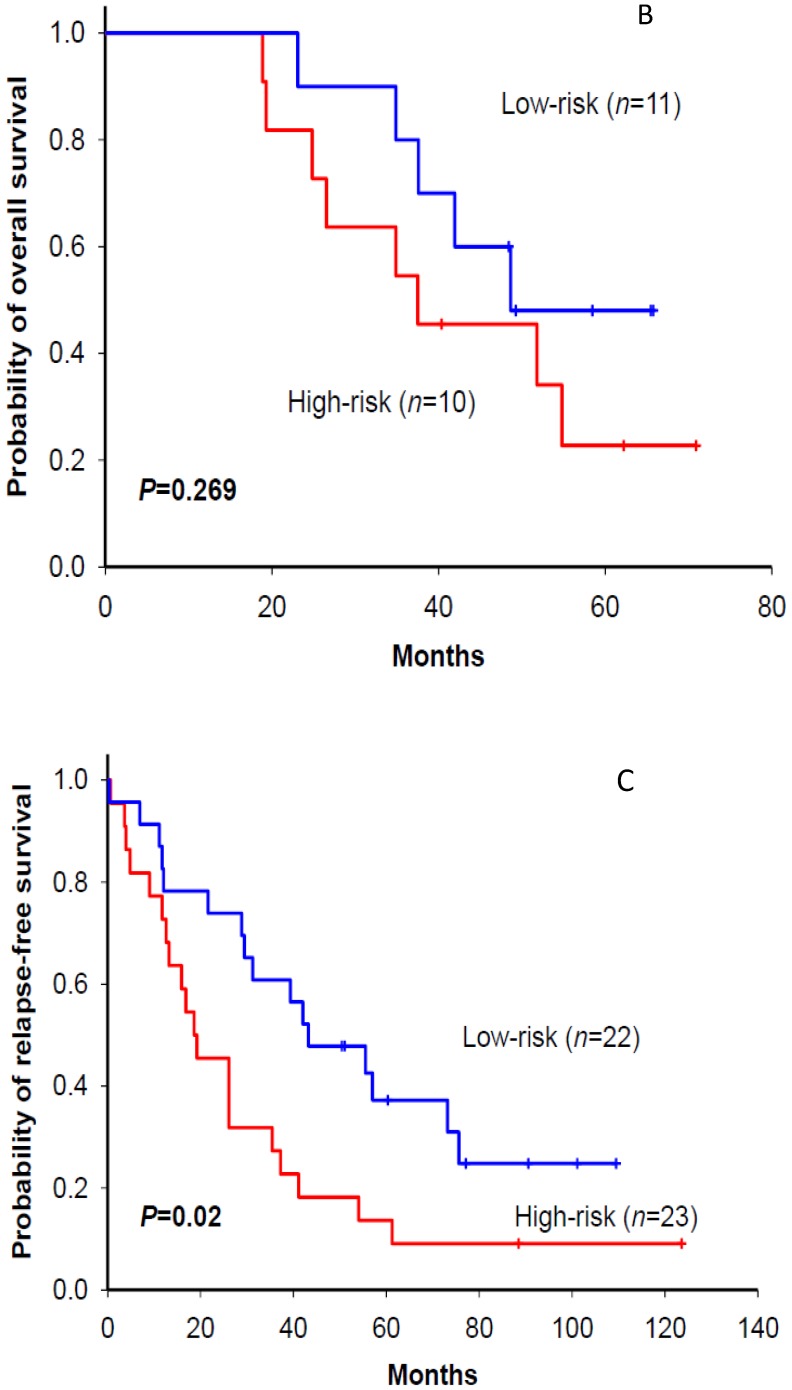

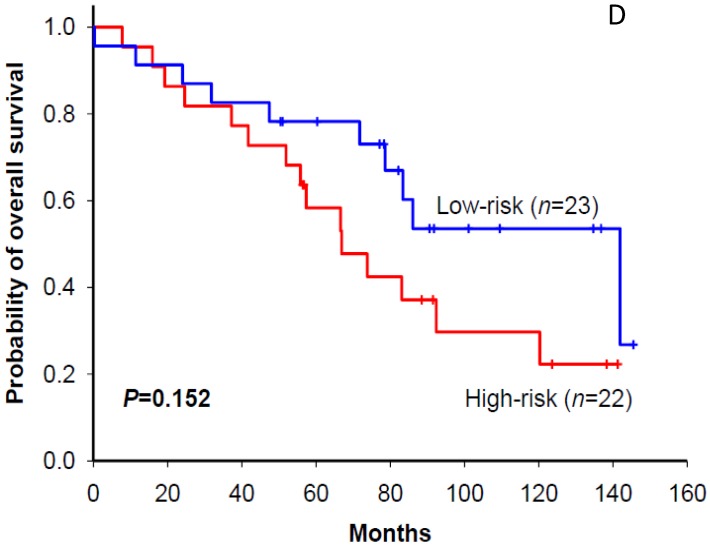
(**A**) Kaplan–Meier relapse-free survival curves of three genes’ copy number variation in 21 CRCLM patients. (**B**) Kaplan–Meier overall survival curves of three genes’ copy number variation in 21 CRCLM patients. (**C**) Kaplan–Meier relapse-free survival curves of three genes’ copy number variation in 45 CRCLM patients. (**D**) Kaplan–Meier overall survival curves of three genes’ copy number variation in 45 CRCLM patients.

We tested the predictive power of the three-gene signature in our CRCLM cohort. We used the average copy number variation of the three genes as the risk score to dichotomize patients into two groups. We found that the low-risk group had a significantly longer relapse-free survival time than the high-risk group in the 21 CRCLM patients (*p* = 0.04, Figure 6A), although this score was insignificant for predicting overall survival (*p* = 0.27, Figure 6B). A final set of three genes—*S100PBP*, *CSMD2*, *TGFBI*—significantly predicted relapse-free survival in our cohort and the public cohort (GSE63490). The low-risk group showed significantly longer distant relapse-free survival in the 45 CRCLM patients (*p* = 0.02, Figure 6C), but it was not significant for predicting the overall survival (*p* = 0.15, Figure 6D). We further conducted multivariate Cox proportional hazard regression analysis with our gene signature and other prognostic factors (including age, gender, and tumor stage) as the predictors. The result shows that the effect of our three-gene signature was significant after adjusting for other factors. The adjusted hazard ratio (HR) was 0.13 (*p* = 0.01) for the 21 CRCLM patients (Table 3).

## 4. Discussion

Many studies have tried to incorporate standard clinical and pathologic parameters of CRCLM patients into clinical risk scores [30,31,32] but failed to validate their results across different patient cohorts. A genomic approach of converting RNA expression levels to risk assessment scores was applied to CRCLM patients after hepatectomy [33]. Balachandran et al. performed a gene expression microarray to develop a 20-gene molecular risk score, and this score can be an independent prognostic biomarker of survival in resected CRCLM. However, DNA CNAs have been demonstrated in colorectal cancer, and RNA degradation is a problem during the handling of tissue samples. Therefore, we used genome-wide DNA CNA profiles from 21 paired normal liver and metastatic CRCLM tumors and identified a three-gene signature prognostic for relapse-free survival. Additionally, we validated this signature for 45 post-hepatectomy CRCLM patients. S100PBP (S100P binding protein) was originally identified by its interaction with S100 calcium-binding protein P. The expression of S100PBP has been reported to be associated with pancreatic ductal adenocarcinoma. The expression of S100PBP shows a negative correlation to that of the metastasis-associated protein S100P [34]. The loss of S100PBP may result in the increased invasion of pancreatic cancer cell lines [35]. CSMD2 (CUB and sushi multiple domain protein 2) was identified as a tumor suppressor for colorectal cancer and used as a predictor of colorectal cancer progress [36]. TGFBI (transforming growth factor-beta induced) was shown to play a role in cell–collagen interactions and was induced by transforming growth factor-beta (TGFB) modulating cell adhesion [37]. TGFBI-expressing cells were found to inhibit tumor cell invasion through the downregulation of MMP-2 and MMP-9 in lung and breast tumor cells [38]. These are consistent with our finding that the loss of S100PBP, CSMD2, and TGFBI might facilitate metastatic spread.

For CRCLM patients, there are several key goals for improving prognosis, including early detection, effective prognostic indicators of treatment response, and accurate identification of patients at high risk for recurrence [8]. It is essential to tailor the therapy according to gene or molecular profiling to avoid unnecessary surgery or treatment-related toxicities without a realized survival benefit. The three-gene signature obtained here may be useful for the development of precision therapy in colorectal cancer with liver metastasis patients. Prognostic and predictive gene signatures have been reported in many malignant cancers. For colorectal cancer patients, few validated prognostic gene signatures have reported assessing outcomes after hepatectomy in colorectal liver metastases (CRCLM) patients. Our three-gene signature was developed using a whole-genome CGH array and had a good prognostic position for the relapse-free survival of CRCLM patients after hepatectomy. Ours is the first DNA copy number alteration (CNA) validated to predict outcomes in CRCLM patients after hepatectomy, thus validating the above-described three-gene signature to assess risk in CRCLM patients treated with hepatectomy.

## Figures and Tables

**Table 1:** Clinical characteristics of the colorectal liver metastases (CRCLM) cohort.

No.	Sex	AGE	Synchronous Metastasis	Relapse Status	Multi-Focal	CEA Elevation	LN	TMN Stage	Primary Site
CRCLM-01	F	73	Yes	Relapse	No	Yes	3	IVA	transverse
CRCLM-02	M	43	No	Relapse	No	No	2	IIIB	rectum
CRCLM-03	M	50	Yes	Relapse	No	No	1	IVA	descending
CRCLM-04	M	83	Yes	No	Yes	Yes	0	IVA	rectosigmoid
CRCLM-05	M	71	No	No	No	No	1	IIIB	cecum
CRCLM-06	M	69	No	Relapse	No	No	0	IIA	rectum
CRCLM-07	F	69	Yes	Relapse	Yes	No	12	IVA	transverse
CRCLM-08	M	67	Yes	Relapse	Yes	Yes	7	IVA	rectum
CRCLM-09	F	60	Yes	No	Yes	Yes	0	IVA	ascending
CRCLM-10	M	61	No	No	Yes	Yes	7	IIIC	rectum
CRCLM-11	F	45	Yes	Relapse	Yes	Yes	4	IVA	sigmoid
CRCLM-12	F	70	Yes	No	Yes	Yes	7	IVA	rectosigmoid
CRCLM-13	F	57	Yes	Relapse	No	Yes	5	IVA	ascending
CRCLM-14	M	66	Yes	No	Yes	Yes	0	IVA	sigmoid
CRCLM-15	M	46	No	No	Yes	No	0	IIA	rectum
CRCLM-17	F	47	Yes	No	No	No	4	IVA	rectum
CRCLM-18	F	39	Yes	Relapse	No	No	1	IVA	rectum
CRCLM-19	M	58	Yes	Relapse	No	Yes	15	IVA	rectum
CRCLM-20	M	73	No	Relapse	No	No	0	IIA	ascending
CRCLM-22	M	63	No	Relapse	No	No	3	IIIB	rectum
CRCLM-23	F	69	No	Relapse	No	Yes	3	IIIB	sigmoid

**Table 2:** Biological functions associated with CRCLM.

Top Functions	*p*-Value	Focus Genes
Cancer	1.24 × 10^−9^ to 2.18 × 10^−2^	159
Gastrointestinal Disease	1.24 × 10^−9^ to 1.57 × 10^−2^	153
Organismal Injury and Abnormalities	1.24 × 10^−9^ to 2.29 × 10^−2^	160
Hepatic System Disease	5.27 × 10^−9^ to 3.5 × 10^−3^	126
Dermatological Diseases and Conditions	1.75 × 10^−8^ to 2.29 × 10^−2^	104

**Table 3:** Multivariate Cox regression analysis for 21 CRCLM patients.

	Hazard Ratio	95% CI	*p*-Value
CRCLM(*n* = 21)			
3-gene signature	0.13	0.03 to 0.61	9.78 × 10^−3^
AGE(cutoff: 60)	0.32	0.09 to 1.22	9.53 × 10^−2^
STAGE(2 vs 3,4)	0.82	0.15 to 4.53	8.19 × 10^−1^
GENDER	2.84	0.71 to 11.29	1.38 × 10^−1^

## Supplementary Materials

The following are available online at http://www.mdpi.com/2077-0383/7/11/446/s1, Figure S1: The circos plot of seven clinical variables. Red: gain; Blue: loss. Circular tracks from outside to inside: primary site: rectum vs others; TMN stage: 3, 4 vs 2; LN: >0 vs 0; CEA elevation: y vs n; multifocal vs N; synchronous meta: y vs n; recurrence: y vs n. Figure S2: The colorectal cancer metastasis signaling pathway. Blue: Relapse and synchronous metastasis-associated probes, Table S1: Two hotspot regions of recurrence-associated candidate genes in 21 CRCLM patients.
