# Supplementary material for: Genome-Wide Scan for Copy Number Alteration Association with Relapse-Free Survival in Colorectal Cancer with Liver Metastasis Patients
Source: J Clin Med. 2018 Nov 18;7(11):446. doi: 10.3390/jcm7110446 (PMC6262537; doi:10.3390/jcm7110446)
Supplement: Supplementary file 1 [file jcm-07-00446-s001.pdf]

J. Clin. Med. **2018**, *7*, 446; doi:10.3390/jcm7110446

Table S1:

Two hotspot regions of recurrence-associated candidate genes in 21 CRCLM patients.

chromosome	Number of gene-harboring probes	Significant	Fisher's p-value
**1**	2067	139(6.72%)	****1.76E-17**
**2**	1896	61(3.22%)	3.99E-01
**3**	1639	47(2.87%)	7.25E-01
**4**	1294	7(0.54%)	1.00E+00
**5**	1258	134(10.65%)	****4.32E-34**
**6**	1353	11(0.81%)	1.00E+00
**7**	1316	6(0.46%)	1.00E+00
**8**	1099	6(0.55%)	1.00E+00
**9**	924	38(4.11%)	5.20E-02
**10**	1135	73(6.43%)	***1.09E-08**
**11**	1139	16(1.4%)	9.99E-01
**12**	1167	35(3%)	6.00E-01
**13**	626	11(1.76%)	9.85E-01
**14**	713	5(0.7%)	9.99E-01
**15**	743	32(4.31%)	***4.40E-02**
**16**	657	21(3.2%)	4.74E-01
**17**	793	19(2.4%)	8.94E-01
**18**	578	35(6.06%)	***2.39E-04**
**19**	588	1(0.17%)	1.00E+00
**20**	564	3(0.53%)	9.99E-01
**21**	266	5(1.88%)	9.12E-01
**22**	358	3(0.84%)	9.98E-01
**X**	929	9(0.97%)	9.99E-01
**Y**	51	0(0%)	1.00E+00

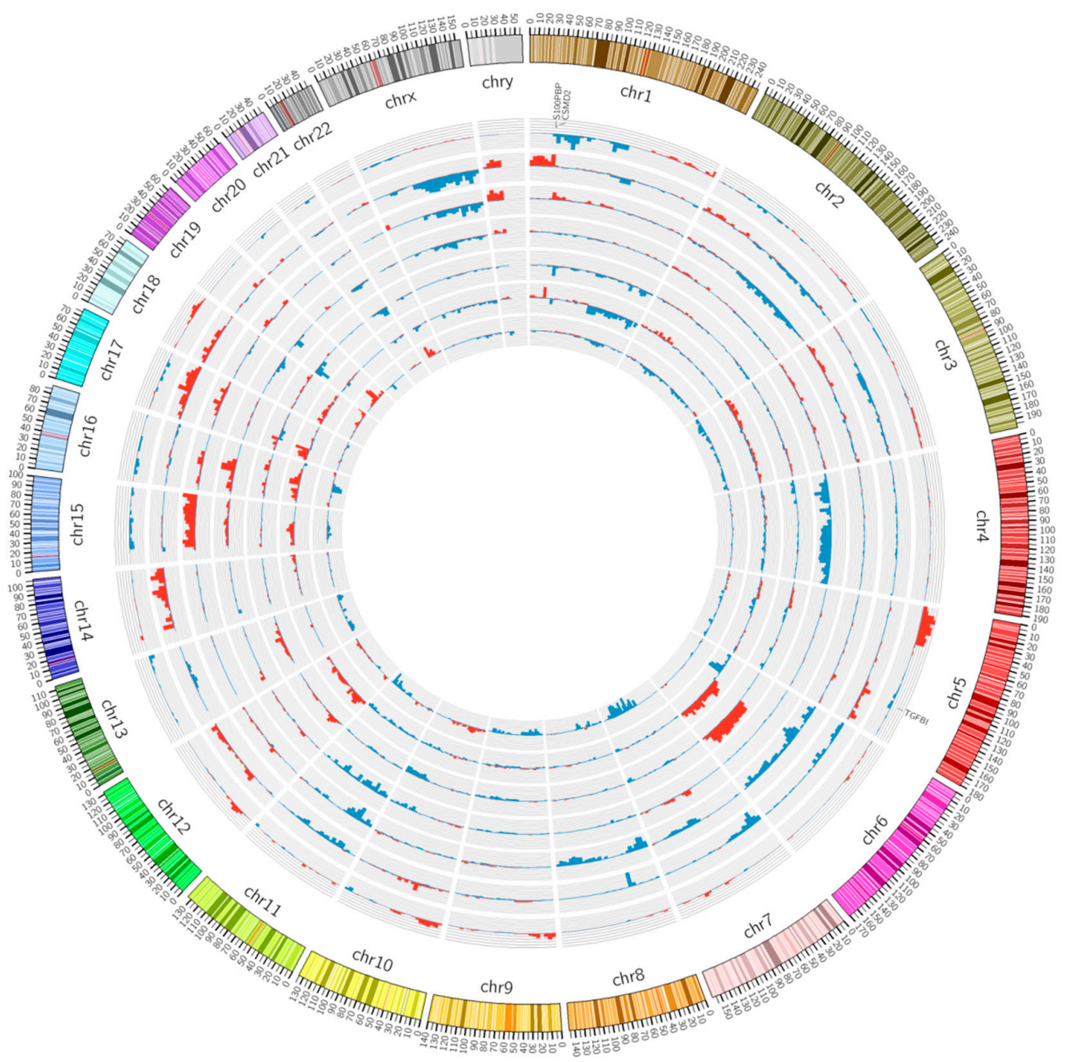

Figure S1: The circos plot of seven clinical variables. Red: gain; blue: loss. Circular tracks from outside to inside: primary site: rectum vs others; TMN stage: 3,4 vs 2; LN: >0 vs 0; CEA elevation: y vs n; multifocal: multifocal vs N; synchronous meta: y vs n; recurrence: y vs n.

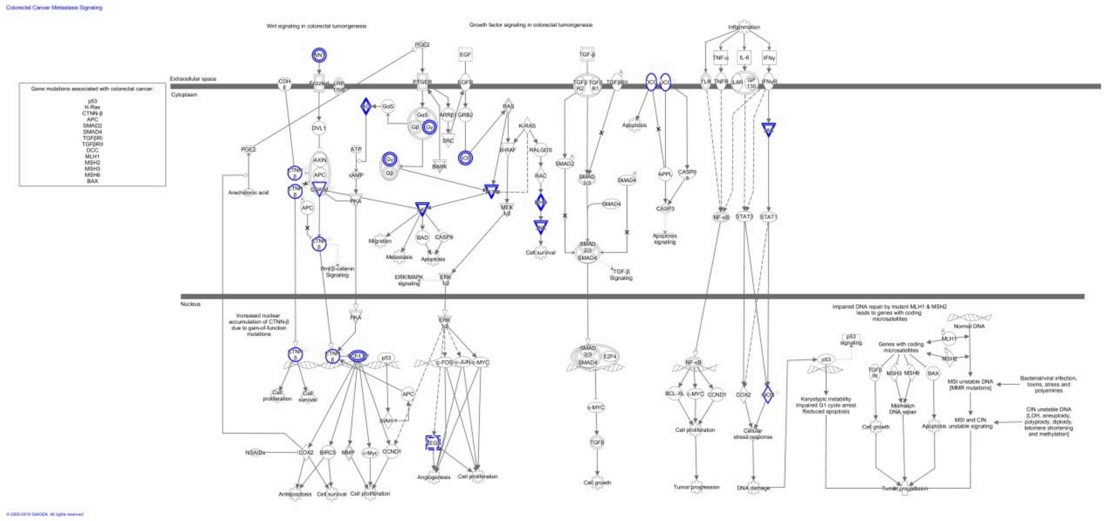

Figure S2: The colorectal cancer metastasis signaling pathway. Blue: Relapse and synchronous metastasis-associated probes.

Supplementary methods:

Copy number variation in clinical variables

To find the probes with differential CNA between the clinical variables, we performed Students' t-test two categories of the following variables: 1. Primary site (rectum vs others) 2. TMN stage (stage 3 and 4 vs stage 2), 3. Number of lymph node metastasis (greater than 0 vs 0) 4. CEA elevation (yes vs no) 5. Multifocal (yes vs no) 6. Synchronous meta (yes vs no) 7. Recurrence (yes vs no) .

For each 5E6 bp, we computed the t-test significant probe density and the density bar plot is shown by the CIRCOS rings in figure 1.2 and the ring sequence, from inner to outer, is as the same as the t-test sequence mentioned in the previous paragraph. The CIRCOS program was downloaded from http://circos.ca/.
